# Premature Rupture of Membranes and Severe Weather Systems

**DOI:** 10.3389/fphys.2020.00524

**Published:** 2020-05-26

**Authors:** Mackenzie L. Wheeler, Michelle L. Oyen

**Affiliations:** Department of Engineering, East Carolina University, Greenville, NC, United States

**Keywords:** preterm, premature, PROM, chorioamnion, fetal, membranes, rupture, failure

## Abstract

There has long been anecdotal evidence of early labor and delivery in severe weather events leading to preterm birth. In particular, significant barometric pressure changes are associated with hurricanes and bomb cyclones. Some authors have related these low pressure weather events to premature rupture of fetal membranes, hypothesizing that the membranes act as an inflated balloon and respond directly to pressure changes. In this article, the key literature including data supporting this hypothesis is reviewed. A simple numerical model, based on a competition between the driving and resisting forces for fetal membrane rupture, is presented. This model provides a quantitative mechanism for membrane failure in the context of storms with low atmospheric pressure. Other sequelae of severe storms that are unrelated to fetal membrane rupture are also discussed. Labor and delivery in the context of major weather events should be understood in a holistic framework that includes both exogenous and endogenous factors relevant to the pregnant patient.

## 1. Introduction

There have been many articles written in the popular press about a potential association between major weather events, such as hurricanes, and early childbirth (LaFrance, 2016; Blau, 2017; Bolluyt, 2018). There is some evidence in the scientific literature in support of such an association, particularly between low levels of barometric pressure and premature rupture of the fetal membranes (PROM) (Polansky et al., 1985; Akutagawa et al., 2007). In this context, “premature” refers not to the gestational age of the pregnancy (instead that is called “preterm”) but the rupture of the membranes prior to the onset of labor. Other studies have found no association between barometric pressure and PROM (Marks et al., 1983). However, the methodology associated with many articles in this genre has been criticized for being insufficient in terms of patient numbers, weather-related data, inadequate control populations, or other deficiencies.

The current work aims to examine this hypothesis of preterm PROM associated with significant drops in barometric pressure associated with major weather events. A number of key studies in this area will be reviewed in the first section of this paper. The physical mechanics of PROM and barometric pressure change will be elucidated with an analytical model in the following section. This will be followed by discussion and conclusions, emphasizing the need for further study on the relationship between major weather events and late-gestation pregnant women, with a view toward potential intervention via evacuation or watchful and conservative medical management.

## 2. Key Literature

An early study completed at the University of Iowa Hospital and published in 1985 (Polansky et al., 1985) showed that PROM occurred more often when the barometric pressure decreased 3 h beforehand. The results of this study further showed that the onset of labor for matched control patients within the same geographical area were not associated with barometric pressure changes. If there was an increase in barometric pressure instead of a drop in pressure, PROM was not affected. This study noted that previous authors examining the issue of PROM and barometric pressure suffered methodological deficiencies in their studies, and that Polansky et al. had designed their study to avoid these flaws. The article postulated that barometric pressure could create a gradient across the chorioamniotic membranes to maintain in utero pressure, but suggested that prostaglandins or other biochemical mechanisms could also be responsible.

A 1997 study focussed on the onset of labor associated with significant decreases in barometric pressure (King et al., 1997). Although not focussed on PROM or membrane rupture, a significant increase in the onset of labor was found in the 24 h after a significant barometric pressure drop and not in the 24 h prior. This study in a journal aimed at nurse-midwives, recommended that low pressure weather systems should be monitored in the context of labor and delivery units and that this association should be mentioned to pregnant women in childbirth classes.

A landmark retrospective study covering 1997–2003 and published in 2007 (Akutagawa et al., 2007) demonstrated that deliveries increased on days with a larger change in barometric pressure in a statistically significant manner. Rupture of the membranes, including premature rupture, was associated with lower barometric pressures, in this study defined by a cut-off value of 758.1 mm Hg (1010.7 hPa in their manuscript). The authors note that labor pains bare associated with both hormones and the autonomic system, and that these both could be affected by local weather and by more general environmental changes. However, they clearly postulate that the membrane rupture and low barometric pressure are not just associated but causal, consistent with the model developed in the following section.

An extensive series of studies of the physical strength of the chorioamnion membrane was performed by Oyen et al. (2004), Oyen et al. (2006), Calvin and Oyen (2007), and Chua and Oyen (2009) showing the decrease in membrane strength with gestational age. These studies focussed on labored versus C-section deliveries and the effects of twin pregnancies, emphasizing endogenous effects. In addition, these authors developed a mechanics-based framework for prediction of preterm birth as a function of changes in pregnancy status, such as polyhydramnios (increased amniotic fluid pressure and volume) or infection within the chorioamnion membrane (Oyen et al., 2004). Here, that mechanics model is applied to consider exogenous factors, in particular the barometric pressure change associated with severe weather events, to test the hypothesis that hurricanes could cause PROM.

## 3. Model and Analysis

A simple model is constructed here for rupture of the fetal (chorioamnion) membrane as a function of substantial decreases in atmospheric pressure. Considerations of fetal membrane rupture will follow that of reference Oyen et al. (2004) where a similar model was examined for endogenous effects such as chorioamnionitis or polyhydramnios. The basis of the model is a competition between stress in and strength of the membrane. In this context, stress is a driving force for mechanical rupture of the membrane, deriving from exogenous causes. In contrast, strength is a resisting force for mechanical failure of the membrane, deriving from endogenous effects. Mechanical failure or rupture of the membrane occurs when the strength of the membrane is exceeded by the applied stress. It is assumed that membrane strength is a function of gestational age alone, and that there is no influence of weather systems on the material properties resisting rupture. In contrast, it is assumed here that the mechanical stress in the membrane is a function of barometric pressure and no consideration of endogenous physiological factors such as decreased membrane strength from infection or changes in other physiological processes is driving mechanical failure. It is entirely likely that the mechanical changes modeled here in isolation are combined with additional contributions to increase the likelihood of PROM in the difficult circumstances of a severe storm. We consider first the membrane strength values based on experimental measurements of puncture force.

**Figure 1:**
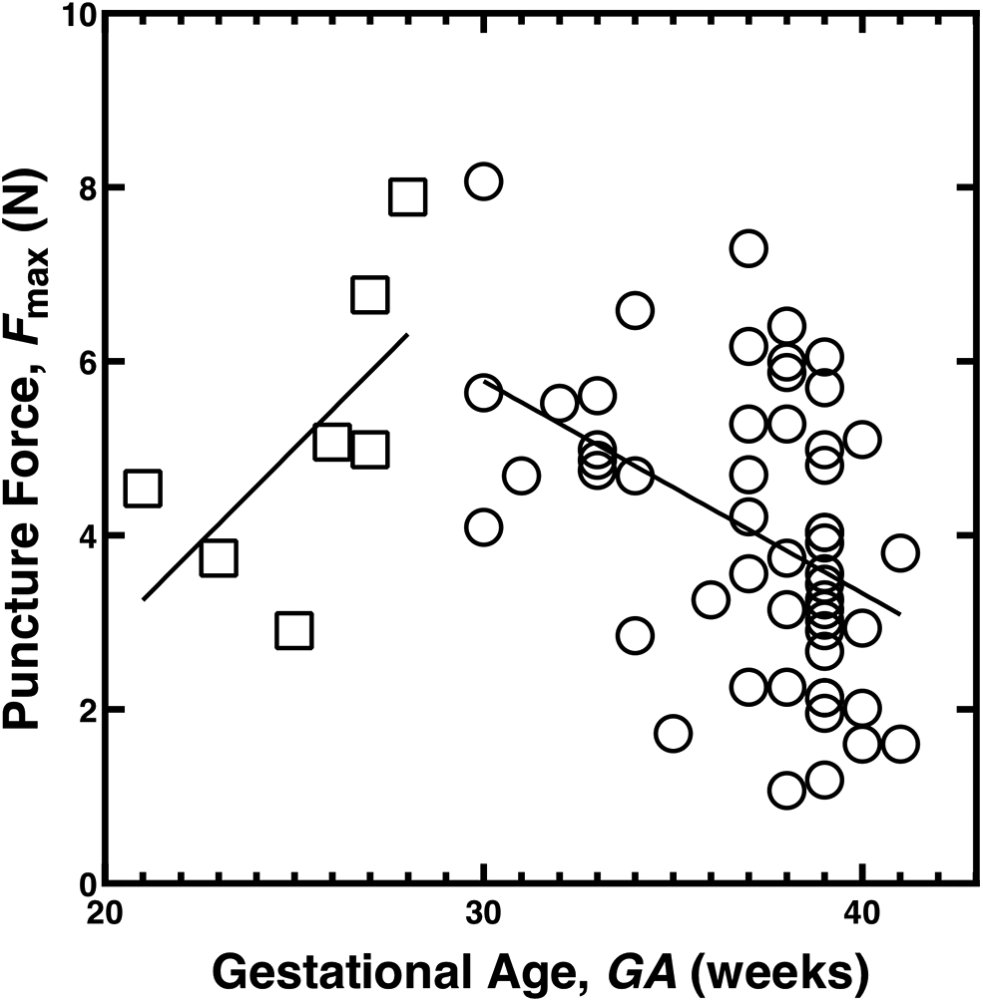
Puncture force for the chorioamnion membrane as a function of gestational age, data from the studies (Oyen et al., 2004, 2006). The data have been split into two groups and fit with linear trend lines, *GA* ≤ 29 weeks (open squares) and *GA*≥30 weeks (open circles).

**Figure 2:**
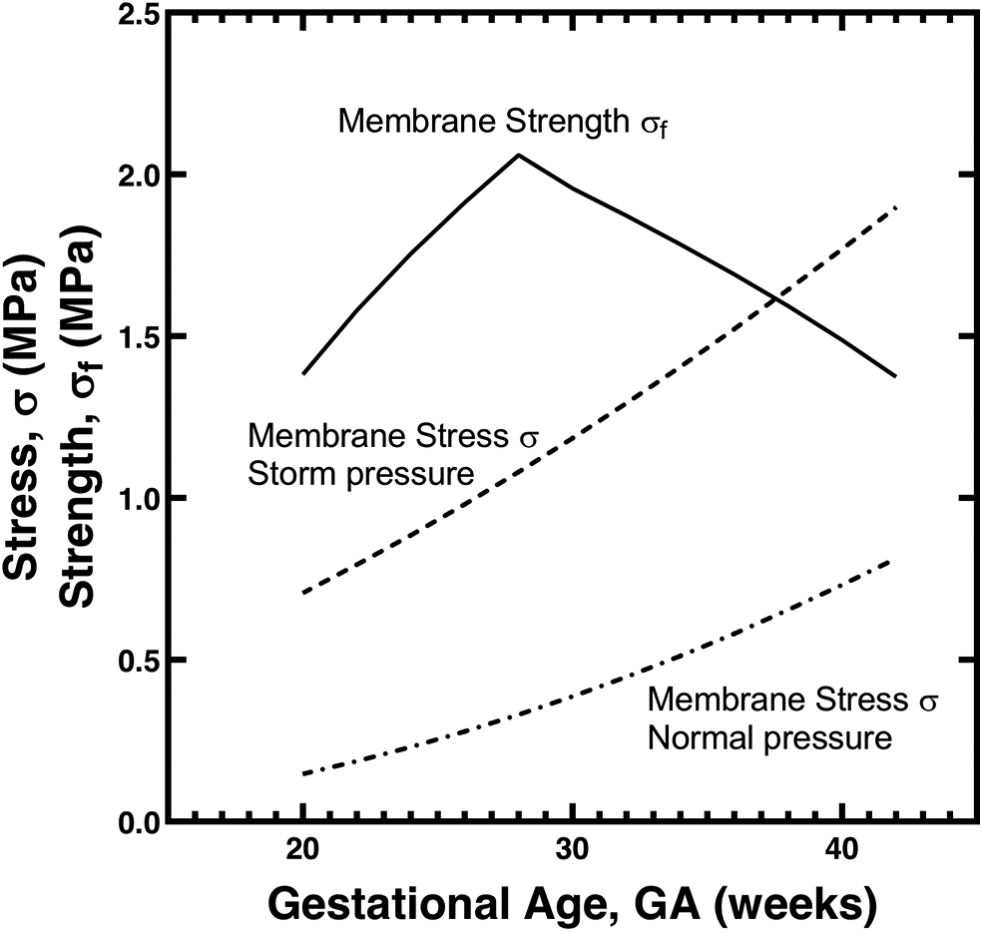
Model for CA membrane stress σ and strength σ_*f*_ as a function of gestational age *GA*. The strength values are calculated from the data in Figure 1 using Equation (1); the stress values for normal and storm atmospheric pressures are calculated from Equation (2) with parameter values as described in the text.

Failure data for membranes were taken from the raw data from the study of puncture force *F*_max_ as a function of gestational age (GA, Oyen et al., 2006). The data were split into two GA groups, *GA* ≤ 29 and *GA*≥30 weeks. The raw data were fit by linear regression for each GA range (Figure 1). These regression lines were used as the input force *F*_max_ as a function of gestational age for calculations of membrane strength, using the equation

(1) $$\begin{array}{r} \sigma_{\text{f}}=\frac{1}{h}{\left(\frac{F_{\text{max}}Eh}{6\pi R}\right)}^{1/2} \end{array}$$

where *h* is the membrane thickness, *E* is the membrane elastic modulus, and *R* = 1.6 mm was the probe radius used in the puncture studies (Oyen et al., 2004, 2006). Here *h* was taken as 250 μm and *E* was taken as 5 MPa based on published data (Helmig et al., 1993). The failure strength σ_f_ shows an increase with GA up to 30 weeks and a decrease thereafter (solid line, Figure 2). We consider next the membrane stress and its changes with barometric pressure.

The membrane stress as a function of fluid pressure was computed according to Laplace's law, as utilized for this context in (Oyen et al., 2004):

(2) $$\begin{array}{r} \sigma =p\frac{r}{2h} \end{array}$$

where *r* is the radius of the sac bounded by the membrane and *h* is again the membrane thickness. The same estimates were used as previously (Oyen et al., 2004) for the size of the sac, linearly increasing from 7 cm at 20 weeks to 13 cm at 40 weeks GA. Amniotic fluid pressure was taken from Weiner et al. (1989) where the data were digitized and the best fit line was taken as *p* = 0.661*GA−5.278. This gives a fluid (gage) pressure of approximately 20 mm Hg above ambient (Faber et al., 1992) at full gestation of GA = 40 weeks (Figure 2, lower dashed line). This baseline was assumed to be for a normal atmospheric pressure of 760 mm Hg. For storm conditions, the atmospheric pressure was assumed to decrease to 730 mm Hg, and thus 30 mm Hg was added to each amniotic fluid pressure due to the change in computing the gage pressure (Figure 2, upper dashed line). It is critical to note that there are no free parameters in this modeling approach, nor are there any pre-factors that are adjusted without mechanism or associated literature data. The calculations of stress and strength are independent save for the shared parameter of membrane thickness (*h*).

The membrane stress is on the order of the membrane strength in the case of normal atmospheric pressure. The value of chorioamnion strength of about 1 MPa near term is consistent with a large review study that found a similar value across a large number of studies and measurement methods (Chua and Oyen, 2009). The stress and strength curves will be shifted slightly up or down depending on the choice of parameter values (*p, E, r, h*) used in the model and on the experimentally-measured force values used (*F*_*max*_). However, the general trend is clear, that a 30 mm Hg drop in atmospheric pressure associated with a storm has the potential to raise the membrane stress significantly, and increase the likelihood that the membrane stress exceeds the membrane strength for a range of late-term gestational ages. Thus, a causal mechanism of membrane rupture due to pressure changes is numerically plausible.

## 4. Discussion

A series of key studies over the course of the last 35 years have demonstrated that there is a clear association of birth with substantial drops in barometric pressure (King et al., 1997), and that this is in particular related to premature rupture of the fetal membranes (Polansky et al., 1985; Akutagawa et al., 2007). A simple model (Oyen et al., 2004) is adapted to consider this association, treating the membranes as a bubble and considering the trade-off between decreasing membrane strength as full term approaches, and increasing membrane stress associated with barometric pressure drops. It is found that a 30 mm Hg pressure drop, consistent with major hurricanes or bomb cyclones (which can occur in winter and not be tropical) is sufficient to provide a mechanistic association between membrane rupture and low atmospheric pressure (Figure 2).

The assumption in the model that atmospheric pressure could influence the stress in the chorioamnion does rely on the membrane being exposed to a pressure difference between the amniotic fluid and the surrounding ambient conditions. It seems more likely that this could be in the case in relatively late gestation when the cervical mucous plug has started to discharge or is missing entirely. This is particularly true if the cervix has also started to dilate, in which case the membranes begin to be directly exposed to ambient air via the vaginal canal.

The model here was plotted (Figure 2) for a pressure drop from a typical atmospheric pressure of 760 mm Hg to a representative storm pressure of 730 mm Hg. This pressure drop of 30 mm Hg in the model was associated with a prediction of membrane rupture at GA = 37.5 weeks at a stress value of 1.62 MPa. A pressure drop of 25 mm Hg, with all other parameters remaining unchanged, predicts rupture at GA = 39 weeks at a stress of 1.54 MPa, a drop of 35 mm Hg predicts rupture at GA = 36.1 weeks at a stress of 1.68 MPa, and a drop of 40 mm Hg predicts rupture at GA = 34.7 weeks at a stress of 1.75 MPa. Thus, a more severe storm in terms of decrease from baseline atmospheric pressure is associated with predictions of membrane rupture at younger gestational ages but greater stresses, since the membrane strength is assumed to decrease with GA (Oyen et al., 2006). These model predictions as noted assume all other model parameters are unchanged, and thus serve as a baseline indicator of the severity of storm pressure changing in isolation of all other potentially changing factors.

Membrane rupture due to low atmospheric pressure is not the only potential mechanism associated with early labor and delivery relative to weather events. Large increases in atmospheric pressure in a relatively short time have also been associated with premature delivery (Akutagawa et al., 2007) although the mechanism for that must be different than the one described herein, perhaps associated with other physiological changes in blood pressure or volume. Less extreme meteorological factors have been associated with both preterm delivery and PROM, including sharp changes in temperature and humidity and strong winds (Yackerson et al., 2008). Such weather conditions were also found to be associated with obstetrical complications such as placental abruption and pre-eclampsia (Yackerson et al., 2007).

There are many possible reasons as to why hurricanes can affect pregnancy, including complications in labor and delivery and subsequent morbidity in the neonate. For example, exposure to hurricanes during a pregnancy can increase the chance of a newborn having to rely on a ventilator (Currie and Rossin-Slater, 2013). The same authors found higher chances of an infant having aspirated meconium, a sign of fetal distress, and that hurricanes were generally associated with low birth weight. There was evidence in this study of significant sequelae in infants even when exposure to storms was early in the pregnancy, as in the first or second trimester (Currie and Rossin-Slater, 2013). Different potential explanations are discussed in this thorough work, including the effect of maternal stress, evacuation out of the path of the storm, and impact of the storm on delivery of medical services in the community. All of these results take on increased importance given our increasing awareness that abnormal conditions surrounding the newborn's arrival can cause poor outcomes for the child not just in the immediate aftermath of birth but also later in life.

One further issue thus far not addressed at all in the literature linking barometric pressure changes and pregnancy is race and ethnicity, and the corresponding health disparities that arise in preterm birth (Culhane and Goldenberg, 2011). The March of Dimes in the US rates states on maternal and infant health and in particular focuses on preterm birth (2019). Many of the states with the worst grades on the March of Dimes scale (2019) coincide with states that are also the most likely to be directly hit in a hurricane (Griggs, 2017). The lack of information linking these two topics presents an interesting opportunity for future research by an interdisciplinary team considering aspects from socioeconomics to biomechanics.

## 5. Conclusion

Labor and delivery in the context of major weather events should be understood in a holistic framework that includes both exogenous and endogenous factors. Management of pregnancies in the path of large storms with significantly low barometric pressures could include education of both health care practitioners and of expectant mothers about this phenomenon. It is entirely likely that evasive action such as evacuation could be warranted for some late-stage pregnancies directly in the path of significant barometric pressure drops on the basis of both the literature and on the basis of the numerical model presented herein. This study motivates further research on this subject, in the context of gathering far more detailed experimental data from regions with high risk for serious weather incidents to better inform expectant mothers and their health care providers. Such research will gain relevance as the occurrence of severe weather events increases due to climate change.

## Data Availability Statement

The dataset analyzed for this study can be found at Oyen (2020) and is related to that in the previous publication (Oyen et al., 2006).

## Author Contributions

MW provided background research in the form of an annotated bibliography and participated in all aspects of writing and editing the manuscript. MO performed the analysis and created the figures in discussion with MW, and participated in all aspects of writing and editing the manuscript.

## Conflict of Interest

The authors declare that the research was conducted in the absence of any commercial or financial relationships that could be construed as a potential conflict of interest.
